# Engineering Reactive Clay Systems by Ground Rubber Replacement and Polyacrylamide Treatment

**DOI:** 10.3390/polym11101675

**Published:** 2019-10-14

**Authors:** Amin Soltani, An Deng, Abbas Taheri, Brendan C. O’Kelly

**Affiliations:** 1 School of Civil, Environmental and Mining Engineering, The University of Adelaide, Adelaide, SA 5005, Australia; An.Deng@adelaide.edu.au (A.D.); Abbas.Taheri@adelaide.edu.au (A.T.); 2 Department of Infrastructure Engineering, Melbourne School of Engineering, The University of Melbourne, Parkville, VIC 3010, Australia; 3 Department of Civil, Structural and Environmental Engineering, Trinity College Dublin, Dublin 2, Ireland; BOKelly@tcd.ie

**Keywords:** expansive clay, polyacrylamide, ground rubber, sediment volume, unconfined compressive strength, swell–shrink capacity, rubber-clustering

## Abstract

This study investigates the combined performance of ground rubber (GR), the additive, and polyacrylamide (PAM), the binder, as a sustainable solution towards ameliorating the inferior geotechnical attributes of an expansive clay. The first phase of the experimental program examined the effects of PAM concentration on the soil’s mechanical properties—consistency, sediment volume attributes, compactability, unconfined compressive strength (UCS), reactivity and microstructure features. The second phase investigated the effects of GR content, with and without the optimum PAM concentration. An increase in PAM beyond 0.2 g/L, the identified optimum concentration, caused the excess PAM to act as a *lubricant* rather than a *flocculant*. This feature facilitated reduced overall resistance to sliding of soil particles relative to each other, thereby adversely influencing the improvement in stress–strain–strength response achieved for ≤0.2 g/L PAM. This transitional mechanism was further verified by the consistency limits and sediment volume properties, both of which exhibited only minor variations beyond 0.2 g/L PAM. The greater the GR content, the higher the mobilized UCS up to 10% GR, beyond which the dominant GR-to-GR interaction (i.e., *rubber-clustering*) adversely influenced the stress–strain–strength response. Reduction in the soil’s swell–shrink capacity, however, was consistently in favor of higher GR contents. Addition of PAM to the GR-blended samples amended the soil aggregate–GR connection interface, thereby achieving further improvements in the soil’s UCS and volume change behaviors. A maximum GR content of 20%, paired with 0.2 g/L PAM, managed to satisfy a major decrease in the swell–shrink capacity while improving the strength-related features, and thus was deemed as the optimum choice.

## 1. Introduction

Clay soils are often characterized as problematic construction materials, as their intrinsic mechanical attributes present significant challenges for geotechnical engineering systems. Meanwhile, shortage of land for development, as well as increasing costs associated with construction and raw materials, necessitate maximum utilization of locally-available materials, one being problematic clay soils. In this context, expansive clays are consistently viewed among the most significant, widespread, costly and least publicized geological hazards, and thus demand further attention [1]. A notable fraction of expansive clays consists of active smectite minerals, which exhibit significant swell–shrink volume changes, as well as desiccation-induced cracking, upon the addition or removal of moisture [2,3]. These adverse actions bring forth major instability concerns to the overlying structures, and thus demand engineering solutions to alleviate the associated socio-economic impacts [4].

The geotechnical engineer can either complete the design within the limitations imposed by the expansive soil or preferably amend the soil’s adverse behaviors by means of physical and/or chemical soil stabilization techniques [5,6]. Physical stabilization practices often involve soil-replacement, pre-wetting, compaction and/or reinforcement. The latter, reinforcement, refers to the placement of randomly-distributed or systematically-engineered geosynthetics, e.g., fibers and geogrids, in the soil regime, thus engendering the development of a spatial three-dimensional reinforcement network in favor of interlocking the soil particles into a unitary mass of induced strength resistance, improved deformability and reduced swell–shrink volume changes [7,8,9,10,11,12,13]. Chemical stabilization refers to the introduction of chemical agents, mainly cementitious binders such as cement and lime, to the soil–water medium, thereby encouraging particle flocculation and hence the development of a dense, uniform matrix coupled with enhanced mechanical properties [10,14,15,16,17,18,19]. In some cases, a combined physical–chemical stabilization scheme may be required to address extreme soil expansivity [20,21,22,23,24]. Although proven effective, conventional stabilization practices often suffer from sustainability issues, attributed to high manufacturing and/or transportation costs, as well as environmental concerns due to greenhouse gas emissions [1]. A sustainable soil stabilization scheme can be characterized as one that maintains a perfect balance between infrastructure performance and the social, economic and ecological processes required to maintain human equity, diversity, and the functionality of natural systems [24]. The transition to *sustainable stabilization* warrants incorporating solid waste materials (e.g., waste tires and textiles, kiln dusts and mine tailings) as an “additive” or “reinforcement” to expansive soils, while opting for non-conventional, environmentally-friendly chemical binders (e.g., polymers, resins and sulfonated oils) for further enhancements.

Discarded tires are among the largest and most problematic sources of solid waste, owing to extensive production and their durability over time [25]. Quite clearly, discarded tire rubbers are not desired at landfills, owing to their low weight-to-volume ratio, resilience, and the fact that they are rarely “flat-packed”. These adverse characteristics, from a landfill perspective, also make them one of the most reusable waste materials for soil stabilization practices, as the rubber is resilient, lightweight, and possesses a rough surface texture. The latter, its rough surface texture, may potentially promote adhesion and/or induce frictional resistance at the soil–rubber interface, and thus alter the soil fabric into a unitary mass of enhanced strength resistance [4,26,27]. The use of recycled tire rubbers in geotechnical engineering dates back to the early 1990s, where theoretical concepts governing the mechanical performance of soil–rubber blends were put into perspective. Much like fiber-reinforced soils, the rubber particles randomly distribute in the soil matrix, and where optimized in content and geometry, enhance the inferior engineering characteristics of the host soil [28,29,30,31,32]. A number of studies have documented the effects of rubber-reinforcement, with and without cementitious binders, on the physical and mechanical behaviors of expansive clays [4,26,27,33,34,35,36,37,38]. Based on these studies, the clay–rubber amending mechanisms can be attributed to the rubber content, with higher contents often producing a more pronounced reduction in the swell–shrink capacity. Moreover, the rubber’s geometrical features, often defined in terms of the rubber’s mean particle size (or *d*_50_) and/or length, have also been reported to play an equally important role.

Much like conventional cementitious binders, *hydrophilic*, *miscible* synthetic polymers, such as polyacrylamides, can be employed to encourage particle flocculation, mainly through clay–polymer interactions, and hence amend the soil fabric into a coherent matrix with enhanced mechanical performance [4,39,40,41,42,43,44,45,46,47]. As the global community increasingly transitions towards sustainable infrastructure construction and development practices, the use of polymeric binders, which often do not have the environmental drawbacks associated with conventional cementitious binders, has gained increased attention. Although commercially branded and readily accessible, such products have generally not yet received widespread acceptance among practicing engineers on account of the lack of sufficient published data by independent establishments, and more importantly, the lack of standard guidelines for effective field implementations [44]. Miscible polymers also possess *lubricant* properties, which reduce the surface tension of water and hence facilitate the movement and sliding of soil particles across each other with much less effort/friction, thereby leading to improved soil compactability [44,48]. Although polymer-treatment appears to have a variety of promising soil amendment properties, the reported results, particularly in the context of geotechnical engineering, are still not consistent towards defining an ad hoc stabilization solution, and as such, further research is urgently required.

This study investigates the combined performance of ground rubber (GR), the additive, and polyacrylamide (PAM), the binder, as a sustainable solution towards ameliorating the inferior geotechnical attributes of an expansive clay. The experimental program was carried out in two phases. The first phase examined the effects of PAM-treatment, at varying PAM concentrations, on the soil’s mechanical properties—consistency limits, sediment volume attributes, compactability, unconfined compressive strength (UCS) and microstructure features; the results of this testing phase were analyzed to identify the optimum PAM concentration. The second phase investigated the effects of GR inclusion, with and without the optimum PAM concentration, through a series of standard Proctor compaction, UCS and soil reactivity tests.

## 2. Materials

### 2.1. Clay Soil

The soil used in this study was sourced from a landfill site located near Adelaide, South Australia; it was reddish-brown in color, and manifested the same typical texture and plasticity features as commonly reported for expansive clays. The physical and mechanical properties of the soil, determined as per relevant ASTM and Australian standards, are outlined in Table 1.

The conventional grain-size analysis indicated a clay fraction (< 2 μm) of 44%, along with 36% silt (2–75 μm) and 20% sand (0.075–4.75 mm). The liquid limit (as determined for 20-mm cone penetration depth using the 80 g–30° fall-cone device) and standard thread-rolling plastic limit were measured as *w*_L_ = 78.04% and *w*_P_ = 22.41%, respectively; giving a plasticity index of *I*_P_ = 55.6%, such that the soil was classified as *clay with high plasticity* (CH) in accordance with the Unified Soil Classification System (USCS). The free swell ratio (FSR)—a quantitative measure of clay mineralogy and hence the soil’s expansive potential [50]—was obtained as FSR = 2.27, thereby indicating that the soil’s clay fraction was mainly dominated by active smectite minerals, such as *montmorillonite*. In terms of expansivity, the FSR corresponded to an undesirable, *high* expansive potential (see Table A1 of the Appendix A section for relevant classification criteria). The standard Proctor compaction test resulted in a relatively high optimum moisture content of *w*_opt_ = 20.24%, along with a maximum dry density of *ρ*_dmax_ = 1.62 Mg/m^3^; the latter produces a minimum void ratio of *e*_min_ = 0.702.

### 2.2. Ground Rubber

Commercially-available tire-derived ground rubber (GR) was used as the additive to partially replace the low-grade host soil. The physical properties and chemical composition of GR, as supplied by the manufacturer or independently measured as per relevant ASTM standards, are summarized in Table 2. In terms of gradation, the rubber particles were found to be similar in size to fine–medium sand (0.075–2 mm). The GR particle diameters corresponding to 10%, 30% and 60% finer—*d*_10_, *d*_30_ and *d*_60_—were obtained as 0.182 mm, 0.334 mm and 0.513 mm, respectively. The coefficients of uniformity and curvature were hence calculated as *C*_u_ = *d*_60_/*d*_10_ = 2.81 and *C*_c_ = *d*_30_^2^/*d*_60_*d*_10_ = 1.20, from which the gradation of the GR material can be classified as equivalent to *poorly-graded sand* (SP) according to the USCS criterion. Other physical properties included a specific surface area of SSA = 0.05 m^2^/g and a specific gravity of *G*_s_^GR^ = 1.09; the latter is approximately 2.5-fold lower than that of the clay soil (*G*_s_^S^ = 2.76). The chemical composition of GR was mainly dominated by styrene–butadiene copolymer and carbon black, with mass fractions of 55% and 25–35%, respectively.

**Figure 1:**
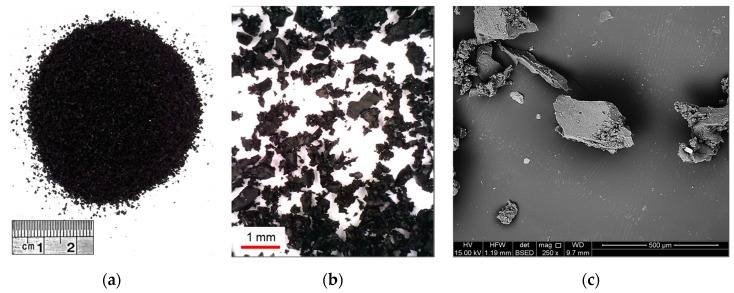
GR material at various magnification ratios: (**a**) 1× magnification; (**b**) 50× magnification (via optical microscopy); and (**c**) 250× magnification (via SEM).

Optical and scanning electron microscopy (SEM) techniques were employed to observe the morphological features of the GR particles, and the results are illustrated in Figure 1. The GR particles were found to be non-spherical and highly-irregular in shape (see Figure 1b); their surfaces encompassed a series of peaks and troughs of varying heights, depths and spacing, as well as occasional cavities and micro-cracks, thus signifying a dominant *rough surface texture* (see Figure 1c). Such morphological features may potentially promote adhesion and/or generate frictional resistance at the soil aggregate–GR interface, and thus alter the soil fabric into a unitary mass of enhanced shear resistance [4,26,27].

### 2.3. Polyacrylamide

**Figure 2:**
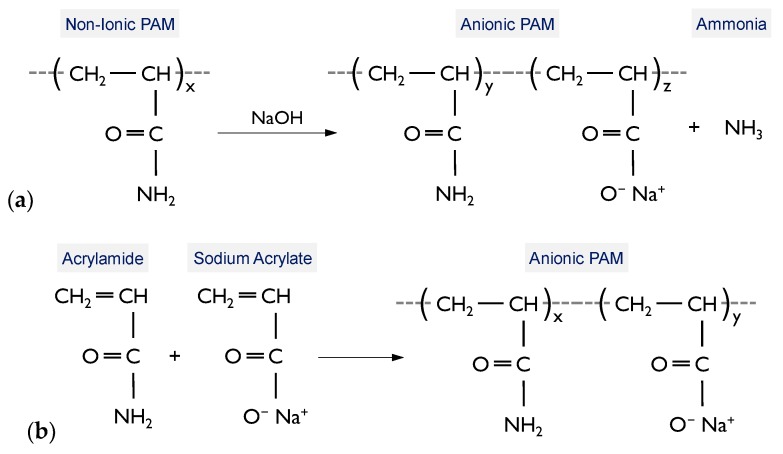
Pathways to anionic PAM formation: (**a**) Hydrolysis [52]; and (**b**) Copolymerization [52].

**Figure A1:**
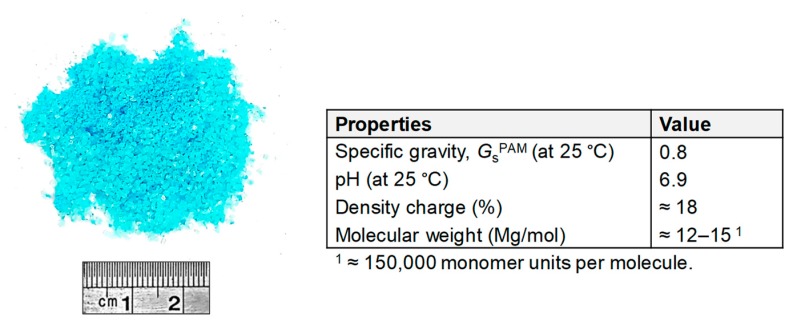
Anionic PAM in its granular form.

A commercially-available polymer agent, chemically referred to as polyacrylamide (PAM), was used as the binder. It was supplied in granular form and was diluted with water for application (see Figure A1 of the Appendix A section). PAMs are a group of *hydrophilic*, *miscible* synthetic polymers formed by the polymerization of acrylamide (AMD) and related monomers (CH_2_=CHC(O)NH_2_); they can be synthesized in non-ionic, cationic or anionic forms [51]. The anionic variant, as used in the present study, can be developed through two common pathways: (i) *hydrolysis* of non-ionic PAM with a strong base such as sodium hydroxide (NaOH), as demonstrated in Figure 2a; and (ii) *copolymerization* of AMD and acrylic acid or a salt of acrylic acid (e.g., sodium acrylate), as illustrated in Figure 2b [52].

Anionic PAMs are mainly employed to encourage flocculation of aqueous suspensions [51,53,54]. Other common applications, as reported in the literature, include their widespread use in the mining industry for thickening and dewatering of concentrates and tailings, as well as their successful adoption in routine construction practices, such as soil compaction, and for erosion control [52,55,56]. The physical and chemical properties of the used PAM, as supplied by the manufacturer, included a pH of 6.9 (at 25 °C), a moderate density charge of approximately 18%, and a relatively high molecular weight of 12–15 Mg/mol (equivalent to approximately 150,000 monomer units per molecule).

## 3. Experimental Program

### 3.1. Mix Designs and Sample Preparations

In this study, a total of thirteen mix designs, consisting of one control (hereafter the natural soil), four PAM-treated, four GR-blended and four PAM + GR cases, were examined (see Table 3). For ease of presentation, the following coding system was adopted to designate the various mix designs:

(1) $$SP_{x}R_{y}$$

where *S* = the natural soil; *P_x_* = *x* g/L PAM; and *R_y_* = *y*% GR.

As outlined in Table 3, the experimental program was carried out in two phases. The first phase, the results of which are presented in Section 4.1, examined the effects of PAM-treatment, at varying PAM concentrations, on the soil’s mechanical properties, namely consistency, sediment volume attributes, compactability, UCS and microstructure features; the results of this section were analyzed to identify the optimum PAM concentration. The second phase, the results of which are presented in Section 4.2, investigated the effects of GR inclusion, with and without the optimum PAM concentration (later to be found as *P*_c_ = 0.2 g/L), through a series of standard Proctor compaction, UCS and soil reactivity tests. The PAM, GR and moisture contents with respect to the various mix designs were, respectively, defined as:

(2) $$(g/L)\;P_{c}=\frac{M_{\mathrm{PAM}}}{V_{W}}$$

(3) $$(\%)\;R_{c}=\frac{M_{\mathrm{GR}}}{M_{S}}\times 100$$

(4) $$(\%)\;w_{c}=\frac{M_{W}\;\mathrm{or}\;M_{\mathrm{PAM}}^{*}}{M_{S}+M_{\mathrm{GR}}}\times 100$$

where *P*_c_ = PAM concentration (g/L); *R*_c_ = GR content (%); *w*_c_ = moisture content (%); *M*_PAM_ = mass of PAM solids; *M*_GR_ = mass of GR particles; *M*_S_ = mass of soil solids; M^*^_PAM_ = mass of PAM solution; *M*_W_ = mass of water; and *V*_W_ = volume of water.

Furthermore, the specific gravity of solids for those mix designs containing GR—*SP_x_R_y_* where *x* = {0, 0.2}, and *y* = {5, 10, 20, 30}—was calculated by the following theoretical relationship [34,57]:

(5) $${G_{s}}^{M}=\frac{{G_{s}}^{S}{G_{s}}^{\mathrm{GR}}(M_{S}+M_{\mathrm{GR}})}{M_{S}{G_{s}}^{\mathrm{GR}}+M_{\mathrm{GR}}{G_{s}}^{S}}$$

where *G*_s_^M^ = specific gravity of GR-blended mixtures (either with or without PAM); *G*_s_^S^ = specific gravity of soil solids (= 2.76); and *G*_s_^GR^ = specific gravity of GR particles (= 1.09).

The natural soil and GR were blended in dry form, as per the selected mix designs outlined in Table 3. The required volume of moisture, either water or PAM solution, corresponding to the mixtures’ standard Proctor optimum moisture content (values presented in Table 6 and Figure 6), was added to each blend and thoroughly mixed using a hand trowel. For those mix designs containing PAM, great care was taken to pulverize any clumped particles, thus targeting homogeneity of the mixtures. A special stainless-steel split mold, as previously described by the authors, was used for sample preparations by means of the *static compaction* technique [4,38]. The mold consisted of three segments—the top collar, the middle section, and the bottom collar. The middle section, measuring 50 mm in diameter and 100 mm in height, accommodates the samples prepared for the UCS tests (see Section 3.2), as well as the core shrinkage tests (a component of the soil reactivity test, as outlined in Section 3.3). The samples were formed by static compaction in the mold in five equal-height layers, each layer having achieved its respective standard Proctor maximum dry density (values presented in Table 6 and Figure 6). Samples for the oedometer swell tests, a second component of the soil reactivity test (see Section 3.3), were prepared in a similar fashion; in this case, however, a different mold with a middle section measuring 50 mm in diameter and 20 mm in height, along with three compaction layers, were adopted. The samples with PAM as the sole stabilizing agent were enclosed in multiple layers of cling wrap and maintained at ambient laboratory temperature conditions for *T*_c_ = 1, 4, 7 and 14 d to examine the effect of curing duration on the UCS. For those mix designs containing the optimum PAM and GR, the same curing procedure was carried out for only 7 d (later to be found as the optimum curing period). The methodologies associated with the UCS, soil reactivity and SEM tests are presented in the following sections.

### 3.2. UCS Test

UCS tests were carried out in accordance with ASTM D2166–16 (American Society for Testing and Materials, PA, USA). The prepared samples were axially compressed at a constant displacement rate of 1 mm/min, as commonly adopted in the literature [26,37,38]. The axial strains and corresponding axial stresses were recorded at predefined time intervals to a point at which the maximum axial stress required for sample failure, the UCS, was achieved. On account of the curing periods adopted for the samples containing PAM, a total of 25 UCS tests—one for control (natural soil), sixteen for PAM-treated, four for GR-blended and four for PAM + GR blends—were carried out to address the thirteen mix designs outlined in Table 3.

### 3.3. Soil Reactivity Test

Soil reactivity tests were carried out in accordance with AS 1289.7.1.1–03 (Standards Australia, NSW, Australia). The test incorporates results obtained from the core shrinkage and the oedometer swell tests, performed on two companion samples, into a unique parameter referred to as the shrink–swell index:

(6) $$I_{\mathrm{ss}}=\frac{\varepsilon_{\mathrm{sh}}+\frac{1}{2}\varepsilon_{\mathrm{sw}}}{\Delta \psi =\psi_{\mathrm{sh}}-\psi_{\mathrm{sw}}}$$

where *I*_ss_ = shrink–swell index (%pF^−1^); [pF] = 1 + log[kPa]; *ε*_sh_ = shrinkage potential (%); *ε*_sw_ = swelling potential (%); ∆*ψ* = range of total suction change (pF); *ψ*_sh_ = total suction upon completion of the core shrinkage test (pF); and *ψ*_sw_ = total suction upon completion of the oedometer swell test (pF).

The methodologies associated with the two test components—the core shrinkage and the oedometer swell tests—are presented in the following.

- **Core shrinkage test:** The prepared 50-mm diameter by 100-mm long samples were allowed to desiccate at ambient laboratory temperature conditions. For each sample, the incurred axial shrinkage strain was regularly monitored (by a dial displacement transducer) to a point at which shrinkage ceased. The desiccated samples were then oven-dried at 105 °C for 24 hr. Final height measurements were taken by a Vernier caliper, from which the ultimate shrinkage strain, denoted as *ε*_sh_ and referred to as the shrinkage potential, was determined.
- **Oedometer swell test:** The prepared 50-mm diameter by 20-mm high samples were inundated with water and allowed to swell in a conventional oedometer setup under a nominal vertical stress of 25 kPa. For each sample, the incurred axial swelling strain was monitored during predefined time intervals to a point at which the ultimate swelling strain, denoted as *ε*_sw_ and referred to as the swelling potential, was achieved.

The shrink–swell index represents percentage axial strain, either shrinkage or swelling, per change in unit suction of the soil, and thus can be employed to estimate free ground surface movements under field conditions [58]. Other applications, as commonly adopted in Australian geotechnical practice, include its use for site classification, as well as its importance to perceive and hence specify the soil degree of expansivity. Classification procedures for expansive soils based on the shrink–swell index, as proposed by Seddon [59], are summarized in Table A2 of the Appendix A section. The denominator in Equation (6), ∆*ψ* = *ψ*_sh_ − *ψ*_sw_, represents the range of total suction change with respect to the sample’s volume increase from air-dry to near saturation conditions; it is commonly taken as ∆*ψ* = 1.8 pF, suggested based on collective experience of the Australian AS 2870 code committee [58]. Although this standard value is widely accepted for natural fine-grained soils, the same may not necessarily hold true for stabilized expansive soils. As such, to examine the validity of the 1.8 pF value for the stabilized expansive soil under investigation in the present study, independent suction measurements were taken upon completion of the core shrinkage and oedometer swell tests by means of the WP4C (METER Group, WA, USA) dew point potentiometer apparatus device [60]. As such, two sets of *I*_ss_ values were measured for each sample: (i) conventional shrink–swell index *I*_ssc_, obtained by ∆*ψ* = 1.8 pF; and (ii) modified shrink–swell index *I*_ssm_, obtained by actual ∆*ψ* measurements. In this regard, as outlined in Table 3, a total of eight mix designs—one for control (natural soil or *SP*_0_*R*_0_), one for PAM-treated (*SP*_0.2_*R*_0_), four for GR-blended (*SP*_0_*R_y_* where *y* = {5, 10, 20, 30}) and four for PAM + GR blends (*SP*_0.2_*R_y_* where *y* = {5, 10, 20, 30})—were examined.

### 3.4. SEM Analysis

The SEM technique was used to observe the evolution of soil fabric in response to PAM-treatment; relevant microstructure analyses were carried out using an SEM characterization scheme developed by Soltani et al. [4]. The Philips XL20 (Amsterdam, The Netherlands) scanning electron microscope was used for SEM imaging; apparatus specifications included a resolution of 4 μm, along with a maximum magnification ratio of 50,000×. In this regard, typical mix designs consisting of *SP*_0_*R*_0_ (natural soil) and *SP*_0.2_*R*_0_ were examined. The desired samples, prepared as per Section 3.1, were first air-dried for approximately 14 d. The desiccated samples were then carefully fractured into cubic-shaped pieces, approximately 1 cm^3^ in volume, and were further scanned at various magnification ratios ranging from 250× to 20,000×.

## 4. Results and Discussion

### 4.1. Phase I: Optimum PAM Concentration

#### 4.1.1. Effect of PAM on Soil Consistency

**Figure 3:**
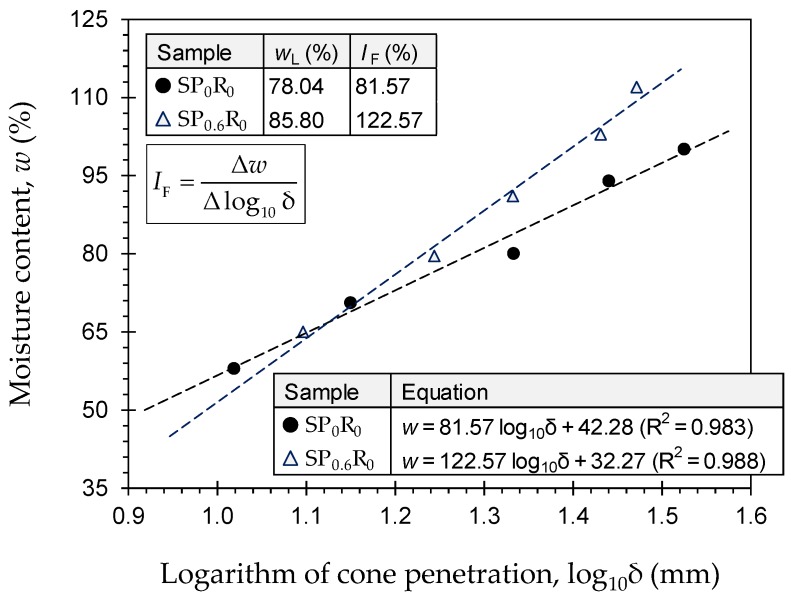
Flow curves for the natural soil (*SP*_0_*R*_0_) and the sample treated with 0.6 g/L PAM (*SP*_0.6_*R*_0_).

Typical flow curves for the natural soil (*SP*_0_*R*_0_) and the sample treated with 0.6 g/L PAM (*SP*_0.6_*R*_0_) are provided in Figure 3. As a result of PAM-treatment, the flow curve exhibited a notable, counterclockwise rotation, thereby leading to a major increase in values of both the flow index *I*_F_ = ∆*w*/∆log_10_δ (where *w* = moisture content, and δ = cone penetration depth) and the liquid limit *w*_L_. Table 4 presents the complete results of the consistency limits tests for the natural soil and various PAM-treated blends—*SP_x_R*_0_ where *x* = {0, 0.1, 0.2, 0.4, 0.6}. The greater the PAM concentration, the higher the consistency limits up to *P*_c_ = 0.2 g/L, beyond which the effect of PAM-treatment was found to be marginal. The rate of increase in the liquid limit, however, was more pronounced compared with that of the plastic limit *w*_P_ and the plasticity index *I*_P_. The natural soil resulted in *w*_L_ = 78.04% (*I*_P_ = 55.63%), while the samples treated with *P*_c_ = 0.1 g/L, 0.2 g/L, 0.4 g/L and 0.6 g/L produced higher values of *w*_L_ = 82.82%, 87.61%, 87.22% and 85.80% (*I*_P_ = 59.09%, 62.76%, 61.36% and 60.93%), respectively. Consequently, PAM-treatment did not alter the soil’s USCS classification, as the original CH “*clay with high plasticity*” category remained unchanged for all treated blends.

The consistency limits, the liquid limit and flow index in particular, can be employed to infer the development of soil fabric [40,61]. An increase in the liquid limit, as is the case with PAM-treated blends (see Table 4), implies that an edge-to-face flocculated fabric dominates the matrix [62]. Compared to a face-to-face dispersed fabric, a flocculated fabric offers higher cone penetration/shear resistance, which in turn promotes higher liquid limits—that is, the standard 20-mm cone penetration depth is achieved for higher moisture contents.

#### 4.1.2. Effect of PAM on Sediment Volume Behavior

Sediment volume features for the soil–water and various soil–PAM suspensions—*SP_x_R*_0_ where *x* = {0, 0.1, 0.2, 0.4, 0.6}—are provided in Table 5. The greater the PAM concentration, the lower the equilibrium sediment volume (and hence the lower the FSR) up to *P*_c_ = 0.2 g/L, beyond which the rate of decrease was marginal. The soil–water suspension (*SP*_0_*R*_0_) had an equilibrium sediment volume of *V*_D_ = 34.0 cm^3^ (FSR = 2.27), whereas the soil–PAM suspensions with *P*_c_ = 0.1 g/L, 0.2 g/L, 0.4 g/L and 0.6 g/L produced lower values of *V*_P_ = 28.5 cm^3^, 25.0 cm^3^, 24.5 cm^3^ and 23.0 cm^3^ (FSR = 1.90, 1.67, 1.63 and 1.53), respectively. As outlined in Table A1, the FSR can be used to perceive the soil degree of expansivity [50]. The natural soil (*SP*_0_*R*_0_) manifested an undesirable, *high* degree of expansivity, while the four PAM-treated cases all exhibited an improved, *moderate* expansive potential.

Much like the consistency limits (see Section 4.1.1), the FSR serves as an indicator of fabric evolution in response to pore-fluid variations, or in this case PAM-treatment [4,40]. A decrease in the FSR for the PAM-treated blends (see Table 5) signifies an increased tendency for particle flocculation, which in turn justifies the observed reduction in the equilibrium sediment volume [40]. In this regard, the maximum tendency for particle flocculation seems to be achieved at *P*_c_ = 0.2 g/L, as greater PAM concentrations did not promote notable further reductions in *V*_P_ and hence the FSR. These trends are also in agreement with those observed for the liquid limit and the flow index, both of which exhibited only minor variations beyond 0.2 g/L PAM.

#### 4.1.3. Effect of PAM on Soil Compactability

**Figure 4:**
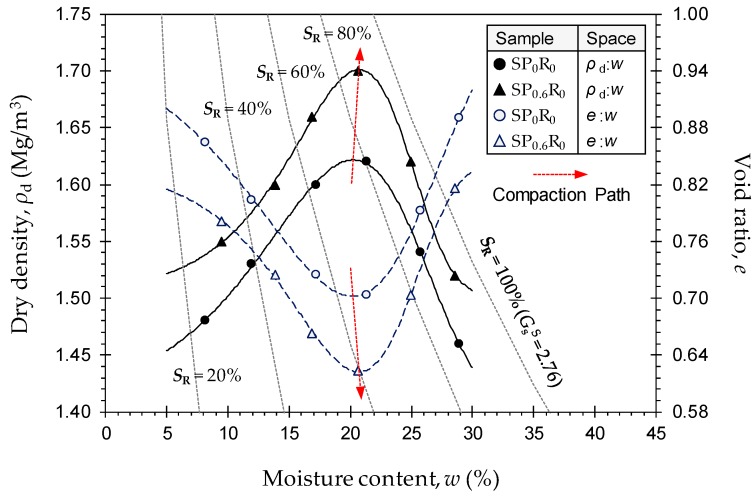
Standard Proctor compaction curves for the natural soil (*SP*_0_*R*_0_) and the sample treated with 0.6 g/L PAM (*SP*_0.6_*R*_0_).

Typical standard Proctor compaction curves, along with representative saturation lines (based on measured *G*_s_^S^ = 2.76), for the natural soil (*SP*_0_*R*_0_) and the sample treated with 0.6 g/L PAM (*SP*_0.6_*R*_0_) are provided in Figure 4. As a result of PAM-treatment, the compaction locus underwent a considerable upward translation in the *ρ*_d_:*w* space (where *ρ*_d_ = dry density, and *w* = moisture content), thereby signifying a notable increase in the maximum dry density *ρ*_dmax_ (follow the “Compaction Path” in Figure 4). However, the variation in corresponding optimum moisture content *w*_opt_ values were found to be marginal. Table 6 presents the complete results of the compaction tests for the natural soil and various PAM-treated blends—*SP_x_R*_0_ where *x* = {0, 0.1, 0.2, 0.4, 0.6}. The natural soil resulted in *ρ*_dmax_ = 1.62 Mg/m^3^ (*w*_opt_ = 20.24%), whereas the samples treated with *P*_c_ = 0.1 g/L, 0.2 g/L, 0.4 g/L and 0.6 g/L produced higher *ρ*_dmax_ values of 1.64 Mg/m^3^, 1.66 Mg/m^3^, 1.67 Mg/m^3^ and 1.70 Mg/m^3^ (*w*_opt_ = 20.28%, 21.06%, 20.20% and 20.72%), respectively.

The increase in maximum dry density with increasing PAM concentration can be attributed to the higher viscosity of PAM solutions compared with that of water. That is, the increase in pore-fluid viscosity for the PAM-treated blends induces *contact lubrication* in the soil matrix; during compaction, this feature facilitates the movement and sliding of soil particles with much less effort/friction, thereby giving rise to higher maximum dry densities [44,48,61].

#### 4.1.4. Effect of PAM on UCS

**Figure 5:**
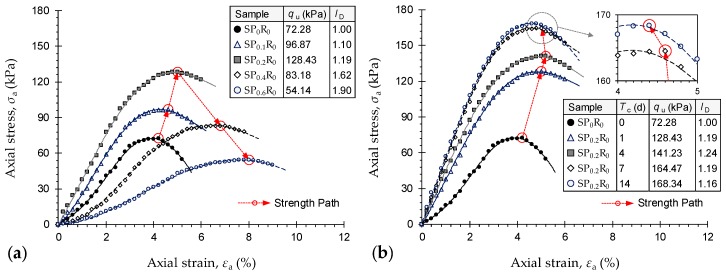
Stress–strain curves for the natural soil and various PAM-treated samples: (**a**) *SP_x_R*_0_, at *T*_c_ = 1 d, where *x* = {0, 0.1, 0.2, 0.4, 0.6}; and (**b**) *SP*_0.2_*R*0 at *T*_c_ = 1, 4, 7 and 14 d.

Stress–strain curves for the natural soil and various PAM-treated samples cured for *T*_c_ = 1 d—*SP_x_R*_0_ where *x* = {0, 0.1, 0.2, 0.4, 0.6}—are provided in Figure 5a. The constitutive response for the natural soil sample (*SP*_0_*R*_0_) demonstrated a rise–fall behavior with a visually-detectable peak point. As a result of increasing PAM concentration, the stress–strain trace progressively transitioned towards a more ductile behavior. In terms of UCS, the greater the PAM concentration, the higher the mobilized UCS up to *P*_c_ = 0.2 g/L, beyond which PAM-treatment was found to adversely influence strength development (follow the “Strength Path” in Figure 5a). At *T*_c_ = 1 d, the natural soil produced a UCS of *q*_u_ = 72.28 kPa, while the addition of 0.1 g/L and 0.2 g/L PAM, at *T*_c_ = 1 d, led to mobilized higher values of 96.87 kPa and 128.43 kPa—equivalent to improvements of *η* = 34% and 78%—respectively. For the same curing period, the higher PAM concentrations of 0.4 g/L and 0.6 g/L resulted in *q*_u_ = 83.18 kPa and 54.14 kPa, respectively; the former, *SP*_0.4_*R*_0_, holding a 15% advantage over the natural soil, whereas the latter, *SP*_0.6_*R*_0_, exhibits a significant 25% reduction with respect to the natural soil.

Stress–strain curves for the natural soil (*SP*_0_*R*_0_) and various samples treated with 0.2 g/L PAM (*SP*_0.2_*R*_0_) cured for *T*_c_ = 1, 4, 7 and 14 d are provided in Figure 5b. For any given PAM concentration, an increase in curing period was found to produce a major increase in the UCS up to *T*_c_ = 7 d, beyond which the effect of curing was found to be marginal (follow the “Strength Path” in Figure 5b), with the axial strain at failure (failure mode) remaining remarkably unchanged. As a typical case, the sample *SP*_0.2_*R*_0_ resulted in *q*_u_ = 128.43 kPa, 141.23 kPa, 164.47 kPa and 168.34 kPa—equivalent to improvements of *η* = 78%, 95%, 128% and 133% with respect to the natural soil (*SP*_0_*R*_0_)—at *T*_c_ = 1, 4, 7 and 14 d, respectively.

The complete results of the UCS tests, along with other stress–strain features, namely the deformability index *I*_D_, the elastic stiffness modulus *E*_50_, the peak strain energy *E*_U_ and the resilient modulus *E*_R_, are summarized in Table 7. At any given curing time, the deformability index—a measure of the sample’s ductility, as defined in the footer of Table 7 [63]—exhibited a monotonically-increasing trend with respect to PAM concentration, meaning that the greater the PAM concentration, the more ductile the sample’s response to unconfined compression. By definition, *I*_D_ has a value of unity for the natural soil (*SP*_0_*R*_0_). As typical cases, the samples *SP*_0.1_*R*_0_, *SP*_0.2_*R*_0_, *SP*_0.4_*R*_0_ and *SP*_0.6_*R*_0_ cured for *T*_c_ = 1 d resulted in *I*_D_ = 1.10, 1.19, 1.62 and 1.90, respectively. For any given PAM concentration, the effect of curing period was found to be marginal; for instance, the sample *SP*_0.2_*R*_0_ resulted in *I*_D_ = 1.19, 1.24, 1.19 and 1.16 at *T*_c_ = 1, 4, 7 and 14 d, respectively. The elastic stiffness modulus, peak strain energy and resilient modulus, as defined in the footer of Table 7 [64,65,66], all demonstrated a trend similar to that observed for the UCS. As such, at any given curing period, the greater the PAM concentration, the higher the *E*_50_, *E*_U_ and *E*_R_ values up to *P*_c_ = 0.2 g/L, beyond which PAM-treatment adversely influenced the sample’s stiffness, toughness and resilience. In terms of stiffness, for instance, the natural soil resulted in *E*_50_ = 1.97 MPa, while the addition of 0.1 g/L, 0.2 g/L, 0.4 g/L and 0.6 g/L PAM at *T*_c_ = 1 d produced *E*_50_ values of 2.83 MPa, 3.67 MPa, 1.39 MPa and 0.75 MPa, respectively. Much like the UCS, the effect of curing period for any given PAM concentration was only notable up to *T*_c_ = 7 d; beyond which the variations in *E*_50_, *E*_U_ and *E*_R_ were negligible. As a typical case, the sample *SP*_0.2_*R*_0_ resulted in *E*_50_ = 3.67 MPa, 4.28 MPa, 5.27 MPa and 5.29 MPa at *T*_c_ = 1, 4, 7 and 14 d, respectively.

### 4.2. Phase II: Combined Effects of Optimum PAM and GR

#### 4.2.1. Effect of PAM + GR on Soil Compactability

**Figure 6:**
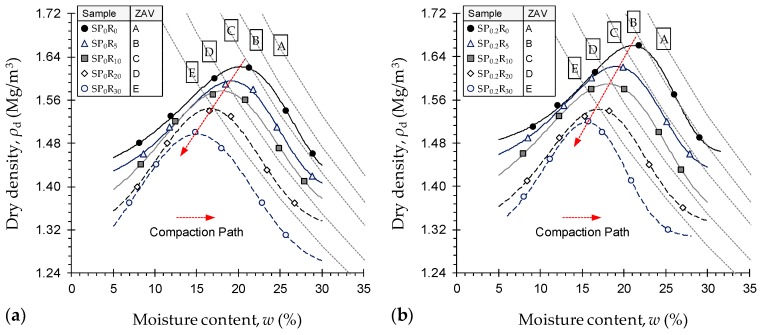
Standard Proctor compaction curves for the natural soil and various GR-blended samples: (**a**) Untreated—*SP*_0_*R_y_* where *y* = {0, 5, 10, 20, 30}; and (**b**) Treated with 0.2 g/L PAM—*SP*_0.2_*R_y_* where *y* = {0, 5, 10, 20, 30}.

Standard Proctor compaction curves, along with corresponding zero-air void (ZAV) saturation lines, for the natural soil and various GR-blended samples untreated—*SP*_0_*R_y_* where *y* = {0, 5, 10, 20, 30}—and treated with 0.2 g/L PAM—*SP*_0.2_*R_y_* where *y* = {0, 5, 10, 20, 30}—are provided in Figure 6a,b, respectively. As a result of GR inclusion, the compaction locus underwent a linear downward–leftward translation over the *ρ*_d_:*w* space, thus indicating a notable decrease in both the maximum dry density and the optimum moisture content associated with increasing GR content (follow the “Compaction Path” in Figure 6). The greater the GR content, the lower the compaction characteristics, with PAM-treated cases holding a slight advantage over their untreated counterparts. The natural soil (*SP*_0_*R*_0_) and the untreated GR-blended samples with *R*_c_ = 5%, 10%, 20% and 30% produced *ρ*_dmax_ = 1.62 Mg/m^3^, 1.60 Mg/m^3^, 1.58 Mg/m^3^, 1.54 Mg/m^3^ and 1.50 Mg/m^3^ (*w*_opt_ = 20.24%, 19.15%, 18.01%, 16.58% and 15.00%), respectively. As a result of PAM-treatment, the aforementioned values were increased marginally to *ρ*_dmax_ = 1.66 Mg/m^3^, 1.62 Mg/m^3^, 1.59 Mg/m^3^, 1.54 Mg/m^3^ and 1.52 Mg/m^3^ (*w*_opt_ = 21.06%, 19.07%, 18.09%, 16.95% and 15.56%), respectively.

Reduction in the compaction characteristics, particularly the maximum dry density, advocates the use of GR as a sustainable, lightweight fill alternative to traditional quarried materials, such as sands and aggregates. The GR material’s lower specific gravity of 1.09 and *hydrophobic* character (<4% water adsorption) compared with that of the soil grains, and active clay minerals in particular, corroborate the observed reduction in the compaction characteristics [36,37,57]. Moreover, the compacted GR particles, though rather resilient, may gradually recover their initial shape due to the elastic rebound, thereby reducing the efficiency of compactive effort and hence leading to lower maximum dry densities [26,57].

#### 4.2.2. Effect of PAM + GR on UCS

**Figure 7:**
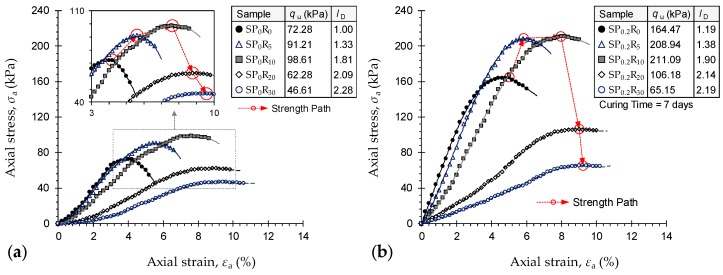
Stress–strain curves for the natural soil and various GR-blended samples: (**a**) Untreated—*SP*_0_*R_y_* where *y* = {0, 5, 10, 20, 30}; and (**b**) Treated with 0.2 g/L PAM at *T*_c_ = 7 d—*SP*_0.2_*R_y_* where *y* = {0, 5, 10, 20, 30}.

Stress–strain curves for the natural soil and various GR-blended samples untreated—*SP*_0_*R_y_* where *y* = {0, 5, 10, 20, 30}—and treated with 0.2 g/L PAM at *T*_c_ = 7 d—*SP*_0.2_*R_y_* where *y* = {0, 5, 10, 20, 30}—are provided in Figure 7a,b, respectively. As a result of increasing GR content, the constitutive response progressively transitioned towards a strain-hardening character; the greater the GR content, the more pronounced the strain-hardening effect and hence the more ductile the failures (see Figure 7a). For any given GR content, the addition of PAM further enhanced the strain–hardening failure mode, while simultaneously enhancing the sample’s UCS and stiffness (see Figure 7b). In terms of UCS, the greater the GR content, the higher the UCS up to *R*_c_ = 10%, beyond which GR inclusion was found to adversely influence strength development (follow the “Strength Path” in Figure 7a). When treated with PAM, the UCS exhibited the same rise–fall behavior with GR content; however, all treated cases outperformed their untreated counterparts (follow the “Strength Path” in Figure 7b). The natural soil (*SP*_0_*R*_0_) resulted in *q*_u_ = 72.28 kPa, while the addition of 5% and 10% GR produced higher values of 91.21 kPa and 98.61 kPa—equivalent to improvements of *η* = 26% and 36%—respectively. The higher GR contents of 20% and 30% resulted in lower values of *q*_u_ = 62.28 kPa and 46.61 kPa, respectively; equating to major reductions of 14% and 36% with respect to the natural soil. As a result of PAM-treatment, the aforementioned values increased to 164.47 kPa, 208.94 kPa, 211.09 kPa, 106.18 kPa and 65.15 kPa, respectively. The latter, *SP*_0.2_*R*_30_, corresponds to a notable 10% UCS reduction with respect to the natural soil, while the former cases, i.e., *SP*_0.2_*R*_0_, *SP*_0.2_*R*_5_, *SP*_0.2_*R*_10_ and *SP*_0.2_*R*_30_, signify major improvements of 128%, 189%, 192% and 47%, respectively.

Other stress–strain features—the deformability index *I*_D_, the elastic stiffness modulus *E*_50_, the peak strain energy (or toughness) *E*_U_ and the resilient modulus *E*_R_—are summarized in Table 8. The greater the GR content, the higher the deformability index and the lower the *E*_50_ value, with PAM-treated cases generally promoting higher values compared with similar untreated cases. Moreover, the peak strain energy and the resilient modulus both demonstrated trends similar to that observed for the UCS. The greater the GR content, the higher the *E*_U_ and *E*_R_ values up to *R*_c_ = 10%, beyond which GR inclusion adversely influenced the sample’s toughness and resilience. Moreover, the samples treated with PAM consistently outperformed their untreated counterparts by exhibiting higher *E*_U_ and *E*_R_ values. In terms of stiffness, for instance, the samples *SP*_0_*R*_0_, *SP*_0_*R*_5_, *SP*_0_*R*_10_, *SP*_0_*R*_20_ and *SP*_0_*R*_30_ resulted in *E*_50_ = 1.97 MPa, 1.96 MPa, 1.51 MPa, 0.72 MPa and 0.47 MPa, respectively. When treated with PAM and cured for *T*_c_ = 7 d, the aforementioned values increased to 5.27 MPa, 4.54 MPa, 3.26 MPa, 1.25 MPa and 0.66 MPa, respectively.

#### 4.2.3. Effect of PAM + GR on Soil Reactivity

**Figure 8:**
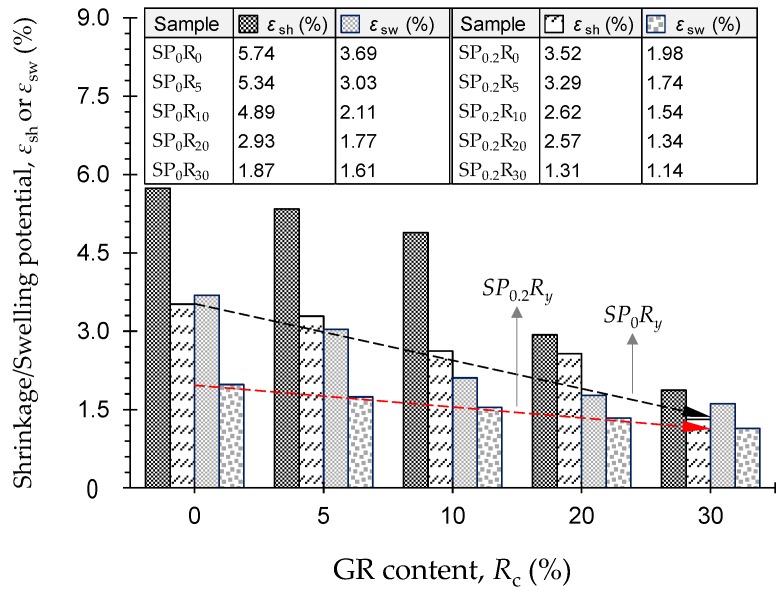
Variations of shrinkage and swelling potentials against GR content for the natural soil and various GR-blended samples—*SP_x_R_y_* where *x* = {0, 0.2}, and *y* = {0, 5, 10, 20, 30}.

Figure 8 illustrates the variations of shrinkage and swelling potentials, *ε*_sh_ and *ε*_sw_, against GR content for the natural soil and various GR-blended samples, untreated and treated with 0.2 g/L PAM—*SP_x_R_y_* where *x* = {0, 0.2}, and *y* = {0, 5, 10, 20, 30}. The greater the GR content, the lower the shrinkage and swelling potentials, following a monotonically-decreasing trend, with PAM-treated cases achieving a notable advantage over their untreated counterparts (compare the trendlines “*SP*_0_*R_y_*” and “*SP*_0.2_*R_y_*” in Figure 8). In terms of swelling, for instance, the natural soil (*SP*_0_*R*_0_) resulted in *ε*_sw_ = 3.69%, while the samples *SP*_0_*R*_5_, *SP*_0_*R*_10_, *SP*_0_*R*_20_ and *SP*_0_*R*_30_ exhibited lower values of 3.03%, 2.11%, 1.77% and 1.61%, respectively. Similar mix designs treated with PAM led to superior performance, as the aforementioned values dropped to 1.98%, 1.74%, 1.54%, 1.34% and 1.14%, respectively.

The complete results of the soil reactivity tests, including the total suction measurements, are summarized in Table 9. As a result of GR inclusion, the initial (or as-compacted) suction *ψ*_o_ exhibited a notable overall increase, with PAM-treated cases promoting higher values compared with similar untreated cases. The natural soil resulted in *ψ*_o_ = 4.73 pF (where [pF] = 1 + log[kPa]), while the addition of 5%, 10%, 20% and 30% GR produced higher values of 4.75 pF, 4.78 pF, 4.77 pF and 4.76 pF, respectively. When treated with PAM, the aforementioned values further increased to 4.74 pF, 4.77 pF, 4.81 pF, 4.79 pF and 4.77 pF, respectively. Unlike the initial suction, both the shrinkage and swelling suctions, *ψ*_sh_ and *ψ*_sw_, were inversely-proportional to the GR content; the effect of PAM, however, was directly- and inversely-proportional to *ψ*_sh_ and *ψ*_sw_, respectively (see Table 9). The natural soil exhibited a total suction change of ∆*ψ* = *ψ*_sh_ − *ψ*_sw_ = 2.07 pF, which is higher than the standard 1.8 pF value commonly adopted in practice. As a result of GR inclusion, ∆*ψ* exhibited a notable increasing trend; it was measured as 2.09 pF, 2.09 pF, 2.28 pF and 2.28 pF for *R*_c_ = 5%, 10%, 20% and 30%, respectively. Similar mix designs treated with PAM produced higher ∆*ψ* values of 2.11 pF, 2.11 pF, 2.14 pF, 2.32 pF and 2.30 pF, respectively. These trends can be attributed to the partial replacement of the *hydrophilic* soil grains with *hydrophobic* GR particles, as well as the lower molding/as-compacted moisture content of various GR-blended samples compared with that of the natural soil (e.g., see Figure 6a). Moreover, the effect of PAM on total suction, particularly for the as-compacted state, can be ascribed to its particle flocculation capacity, as well as the higher molding dry density (or lower void ratio) of PAM-treated samples compared with similar untreated cases (e.g., see Figure 6b).

**Figure 9:**
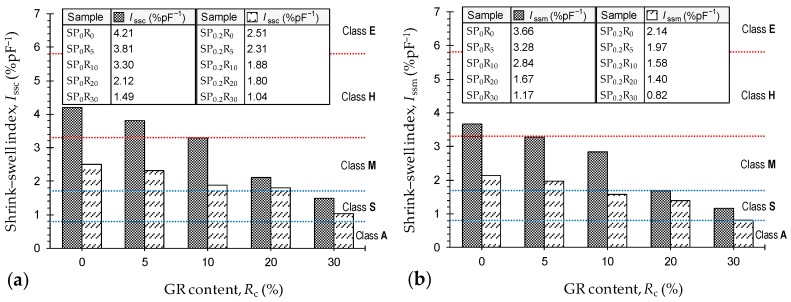
Variations of the shrink–swell index against GR content for the natural soil and various GR-blended samples—*SP_x_R_y_* where *x* = {0, 0.2}, and *y* = {0, 5, 10, 20, 30}: (**a**) Conventional approach *I*_ssc_; and (**b**) Modified approach *I*_ssm_.

Figure 9a,b illustrate the variations of the conventional and modified shrink–swell indices (*I*_ssc_ and *I*_ssm_, respectively) against GR content for the tested samples. As is evident from Table 9, the actual total suction change ∆*ψ* was consistently greater than the standard 1.8 pF value; as such, by definition (see Equation (6)), one can conclude that *I*_ssc_ > *I*_ssm_. The greater the GR content, the lower the shrink–swell indices, following a monotonically-decreasing trend, with PAM-treated cases outperforming similar untreated cases in terms of their lower shrink–swell indices. As typical cases outlined in Figure 9b, the samples *SP*_0_*R*_0_, *SP*_0_*R*_5_, *SP*_0_*R*_10_, *SP*_0_*R*_20_ and *SP*_0_*R*_30_ resulted in *I*_ssm_ = 3.66 %pF^−1^, 3.28 %pF^−1^, 2.84 %pF^−1^, 1.67 %pF^−1^ and 1.17 %pF^−1^, respectively. When treated with PAM, the aforementioned values dropped to 2.14 %pF^−1^, 1.97 %pF^−1^, 1.58 %pF^−1^, 1.40 %pF^−1^ and 0.82 %pF^−1^, respectively.

As demonstrated in Table A2, the shrink–swell index can be used to specify the soil degree of expansivity [59]—corresponding classifications based on the *I*_ssc_ and *I*_ssm_ indices are outlined in Figure 9a,b, respectively. The samples *SP*_0_*R*_0_, *SP*_0_*R*_5_, *SP*_0_*R*_10_, *SP*_0_*R*_20_ and *SP*_0_*R*_30_ were classified as H, H, H–M, M and S based on the *I*_ssc_ index, while consideration of the *I*_ssm_ index for the same samples resulted in improved classifications of H, M, M, S and S, respectively (where H = *highly expansive*, M = *moderately expansive*, and S = *slightly expansive*). As a result of PAM-treatment, the aforementioned classifications were either improved or maintained—M, M, M, M and S based on the *I*_ssc_, and M, M, S, S and S based on the *I*_ssm_. As it stands, the conventional shrink–swell index, computed based on ∆*ψ* = 1.8 pF, tends to overestimate the expansive potential of the test soil and various soil–GR blends, and thus can be deemed as a conservative approach [60]. The modified index, however, offers a more progressive and hence sustainable classification scheme.

### 4.3. Amending Mechanisms

Polymers, for the most part, alter the soil fabric through clay–polymer interactions [51]. Cationic polymers are readily adsorbed onto the negatively-charged clay particle surfaces through Coulombic (or electrostatic) attraction; this process induces localized charge reversal on each clay particle, thereby allowing localized areas with opposite charge between different clay particles to interact and form flocs [67,68]. Neutral (non-ionic) polymers, on the other hand, achieve adsorption through van der Waals and/or hydrogen bonding [67,68,69]. Although clay particles tend to repel anionic polymers due to charge repulsion, which would be the case for the anionic PAM used in this study, attraction can still be achieved through exchangeable, divalent cations in the clay–water medium, e.g., calcium (Ca^2+^) and magnesium (Mg^2+^), which act as so-called “bridges” between the polymer and the clay particle surfaces [53,67,70,71]. The development and propagation of these strong *cationic bridges* between adjacent clay particle surfaces in the matrix lead to a decrease in the thickness of the diffused double layers, which in turn induced aggregation and flocculation of the clay particles and hence favored an enhanced strength performance coupled with a reduced tendency for swell–shrink volume changes. It should be noted that the maximum strength benefits of flocculation are often achieved when polymers, and anionic PAMs in particular, cover a surface area equivalent to half the polymer’s saturation capacity [72]. An increase in polymer concentration beyond this level causes the excess polymer material to act as a *lubricant* rather than a *flocculant*; this feature facilitates the sliding of soil aggregates across each other, and thus may adversely influence certain mechanical attributes, such as the stress–strain–strength response. This mechanism elucidates the observed reduction in the UCS (see Table 7), as well as the minor variations noted in the consistency limits (see Table 4) and the sediment volume features (see Table 5), for PAM concentrations greater than 0.2 g/L.

**Figure 10:**
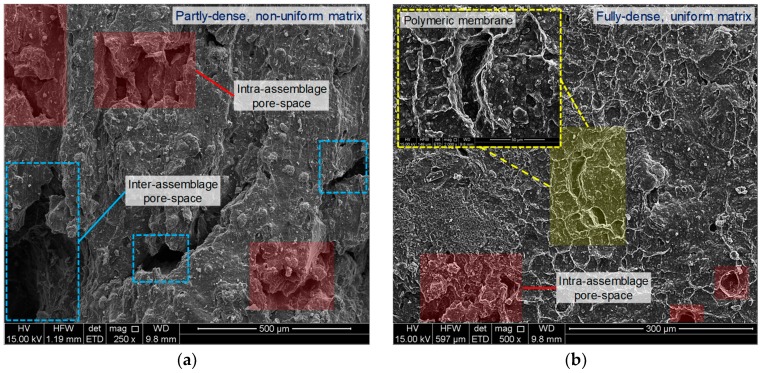
SEM micrographs for the tested samples: (**a**) *SP*_0_*R*_0_; and (**b**) *SP*_0.2_*R*_0_.

Figure 10a,b illustrate typical SEM micrographs for the natural soil (*SP*_0_*R*_0_) and the sample treated with 0.2 g/L PAM (*SP*_0.2_*R*_0_), respectively. The microfabric of the natural soil sample, as outlined in Figure 10a, showed a partly-dense, non-uniform matrix. Moreover, a notable number of large inter- and intra-assemblage pore-spaces developed between and within the soil aggregates, respectively, were found to dominate the soil matrix; these pore-spaces facilitate the entrance of water into the sample during swelling [3]. As a result of PAM-treatment (see Figure 10b), the microfabric became more uniform in nature, signifying aggregation and flocculation of the clay particles (owing to the *cationic bridging mechanism*), and thus the development of a fully-dense matrix with a dominant edge-to-face flocculated fabric. Polymeric membranes were also clearly visible between and within the soil aggregates, which play a major role in reducing the number and extent of the inter- and intra-assemblage pore-spaces, thereby favoring significant improvements in the soil’s UCS and volume change behaviors.

Much like fiber-reinforced soils, the GR inclusions are able to alter the soil fabric through improvements achieved in three aspects: (i) increase in non-expansive content; (ii) frictional resistance generated at soil aggregate–GR interfaces; and (iii) mechanical interlocking of soil and GR particles [1,3,4,26,33,34]. The swell–shrink capacity is primarily a function of the soil’s expansive clay content. An increase in GR content substitutes a larger portion of the expansive clay content with non-plastic, *hydrophobic* rubber particles, thereby leading to a further decrease in the swell–shrink capacity (see Table 9). The generated interfacial frictional resistance is a function of the soil aggregate–GR contact area, with greater contact levels offering a higher shear resistance against external forces. The greater the number of included GR particles (i.e., increase in GR content), the greater the contact levels achieved between the GR particles and soil aggregates, and thus the higher the developed frictional resistance against compression and swell–shrink movements. The mechanical interlocking of soil aggregates and GR particles is achieved during sample preparation (or compaction); it may induce sample adhesion by immobilizing the soil aggregates undergoing shearing, swelling and shrinkage. Quite clearly, the more pronounced the achieved mechanical interlocking, the higher the resistance against compression and swell–shrink movements. As such, this amending mechanism can also be ascribed to the GR content—the greater the GR content, the greater the number of potentially interlocked soil aggregates, and thus the higher the developed resistance against compression, swelling and shrinkage. In practice, however, the latter two amending mechanisms, *interfacial frictional resistance* and *mechanical interlocking*, only hold provided that the GR particles are well distributed in the soil medium—meaning that the GR particles do not cluster during compaction [1,27,36]. At high GR contents, the behavior at some points within the blended sample could be governed by a dominant GR-to-GR interaction; this effect, referred to as *rubber-clustering*, promotes a notable improvement in the sample’s ductility/deformability while offsetting the desired soil-to-GR interaction capable of enhancing its UCS (follow the “Strength Path” in Figure 7a). Such adverse effects were evident for all samples containing 20% and 30% GR, as the previously-improved UCS dropped below that of the natural soil (e.g., compare the samples “*SP*_0_*R*_10_” and “*SP*_0_*R*_20_” in Figure 7a). When treated with PAM, the soil aggregate–GR connection interface can be further improved, as PAM facilitates the embedment of GR particles between the flocculated soil aggregates with increased efficiency; this leads to further improvements in the sample’s UCS and swell–shrink capacity (see Figure 7b and Table 9).

## 5. Concluding Remarks

The present study has arrived at the following conclusions.

- The greater the PAM concentration, the higher the consistency limits and the lower the FSR up to 0.2 g/L PAM, beyond which the effect of PAM-treatment was found to be marginal. Hence, the optimum PAM concentration for particle flocculation was achieved at 0.2 g/L PAM.
- The greater the PAM concentration, the higher the maximum dry density, while the variations in optimum moisture content were rather marginal. An increase in pore-fluid viscosity, as is the case with PAM-treated blends, induces *contact lubrication* in the soil matrix. During compaction, this feature facilitates reduced overall resistance to the movement and sliding of soil particles relative to each other, thereby allowing a closer packing of the soil particles and hence the development of a higher maximum dry density.
- Further, the greater the PAM concentration, the higher the developed UCS, stiffness and toughness up to 0.2 g/L PAM, beyond which PAM-treatment was found to adversely influence these properties. An increase in PAM beyond 0.2 g/L, the optimum concentration, caused the excess PAM to act as a *lubricant* rather than a *flocculant*. During compression, this feature facilitated easier sliding of the soil particles relative to each other, thereby adversely influencing the previously-improved stress–strain–strength response.
- As a result of GR inclusion, both the maximum dry density and the optimum moisture content exhibited a linear, monotonically-decreasing trend with increasing GR content. The reduction in the maximum dry density, in particular, advocates the use of GR as a sustainable, lightweight alternative for common quarried fill materials, such as sands and aggregates.
- Further, the greater the GR content, the higher the developed UCS and toughness up to 10% GR, beyond which the dominant GR-to-GR interaction (i.e., *rubber-clustering*) adversely influenced the blended samples’ UCS and toughness. The sample stiffness, however, exhibited a monotonically-decreasing trend with GR content. Moreover, reduction in the swell–shrink capacity, and thus the degree of expansivity/reactivity, was consistently in favor of higher GR contents.
- The addition of PAM to the GR-blended samples enhanced the soil aggregate–GR connection interface, thereby producing further improvements in the samples’ compactability, stress–strain–strength response and swell–shrink capacity. A maximum GR content of 20%, paired with 0.2 g/L PAM, managed to satisfy a major decrease in the swell–shrink capacity while improving the strength-related features, and thus was deemed as the optimum choice.

## Figures and Tables

**Table 1:** Physical and mechanical properties of the clay soil.

Properties	Value/Description	Standard Designation
Specific gravity of solids, *G*_s_^S^	2.76	ASTM D854–14
Clay fraction [<2 μm] (%)	44	ASTM D422–07
Silt fraction [2–75 μm] (%)	36	ASTM D422–07
Sand fraction [0.075–4.75 mm] (%)	20	ASTM D422–07
Liquid limit, *w*_L_ (%)	78.04	AS 1289.3.9.1–15
Plastic limit, *w*_P_ (%)	22.41	AS 1289.3.2.1–09
Plasticity index, *I*_P_ (%)	55.63	AS 1289.3.3.1–09
Flow index, *I*_F_ (%) ^1^	81.57	Sridharan et al. [49]
USCS classification	CH	ASTM D2487–11
Free swell ratio, FSR ^2^	2.27	Prakash and Sridharan [50]
Dominant clay mineral	Montmorillonite	Prakash and Sridharan [50]
Degree of expansivity	High	Prakash and Sridharan [50]
Optimum moisture content, *w*_opt_ (%)	20.24	ASTM D698–12
Maximum dry density, *ρ*_dmax_ (Mg/m^3^)	1.62	ASTM D698–12

^1^*I*_F_ = ∆*w*/∆log_10_δ (where *w* = moisture content, and δ = cone penetration depth); and ^2^ Ratio of equilibrium sediment volume of 10 g oven-dried soil, passing 425 μm sieve, in distilled water to that of kerosene.

**Table 2:** Physical properties and chemical composition of GR.

Properties	Value/Description	Standard Designation
Specific gravity of solids, *G*_s_^GR^	1.09	—
Particle diameter *d*_10_ (mm)	0.182	ASTM D422–07
Particle diameter *d*_30_ (mm)	0.334	ASTM D422–07
Particle diameter *d*_50_ (mm)	0.478	ASTM D422–07
Particle diameter *d*_60_ (mm)	0.513	ASTM D422–07
Particle diameter *d*_90_ (mm)	0.864	ASTM D422–07
Coefficient of uniformity, *C*_u_	2.81	ASTM D422–07
Coefficient of curvature, *C*_c_	1.20	ASTM D422–07
USCS classification	SP ^1^	ASTM D2487–11
Water adsorption (%)	< 4	—
Softening point (°C)	170	—
Styrene–butadiene copolymer (% by mass)	55	—
Carbon black (% by mass)	25–35	—
Acetone extract (% by mass)	5–20	—
Zinc oxide (% by mass)	2–3	—
Sulphur (% by mass)	1–3	—

^1^ Equivalent to *poorly-graded sand*.

**Table 3:** Soil–PAM–GR mix designs and their properties.

Group	Sample	*P*_c_ (g/L)	*R*_c_ (%)	*G*_s_^M 1^	*T*_c_ (d) ^2^	Tests
Control	*SP*_0_ *R*_0_	0	0	2.76	—	CL ^3^, SV ^4^, SPC ^5^, UCS, SR ^6^, SEM
Phase I PAM-treated	*SP*_0.1_ *R*_0_	0.1	0	2.76	1, 4, 7, 14	CL, SV, SPC, UCS
	*SP*_0.2_ *R*_0_	0.2 ^7^	0	2.76	1, 4, 7, 14	CL, SV, SPC, UCS, SR, SEM
	*SP*_0.4_ *R*_0_	0.4	0	2.76	1, 4, 7, 14	CL, SV, SPC, UCS
	*SP*_0.6_ *R*_0_	0.6	0	2.76	1, 4, 7, 14	CL, SV, SPC, UCS
Phase II GR-blended	*SP*_0_ *R*_5_	0	5	2.57	—	SPC, UCS, SR
	*SP*_0_ *R*_10_	0	10	2.42	—	SPC, UCS, SR
	*SP*_0_ *R*_20_	0	20	2.20	—	SPC, UCS, SR
	*SP*_0_ *R*_30_	0	30	2.04	—	SPC, UCS, SR
Phase II PAM + GR	*SP*_0.2_ *R*_5_	0.2	5	2.57	7 ^8^	SPC, UCS, SR
	*SP*_0.2_ *R*_10_	0.2	10	2.42	7	SPC, UCS, SR
	*SP*_0.2_ *R*_20_	0.2	20	2.20	7	SPC, UCS, SR
	*SP*_0.2_ *R*_30_	0.2	30	2.04	7	SPC, UCS, SR

^1^ Mixture specific gravity, obtained by Equation (5); ^2^ Curing time for the UCS tests; ^3^ Consistency limits tests; ^4^ Sediment volume test; ^5^ Standard Proctor compaction test; ^6^ Soil reactivity test; ^7^ Optimum PAM concentration; and ^8^ Optimum curing time.

**Table 4:** Summary of the consistency limits for the natural soil and various PAM-treated blends—*SP_x_R*_0_ where *x* = {0, 0.1, 0.2, 0.4, 0.6}.

Sample	*w*_L_ (%)	*w*_P_ (%)	*I*_P_ (%)	USCS	*I*_F_ (%)
*SP*_0_ *R*_0_	78.04	22.41	55.63	CH	81.57
*SP*_0.1_ *R*_0_	82.82	23.73	59.09	CH	97.34
*SP*_0.2_ *R*_0_	87.61	24.85	62.76	CH	118.54
*SP*_0.4_ *R*_0_	87.22	25.86	61.36	CH	120.70
*SP*_0.6_ *R*_0_	85.80	24.87	60.93	CH	122.57

**Table 5:** Summary of the sediment volume features for the natural soil and various PAM-treated blends—*SP_x_R*_0_ where *x* = {0, 0.1, 0.2, 0.4, 0.6}.

Sample	*V*_D_ (cm^3^)	*V*_P_ (cm^3^)	*V*_K_ (cm^3^)	FSR	Degree of Expansivity [50]
*SP*_0_ *R*_0_	34.0	—	15.0	2.27	High
*SP*_0.1_ *R*_0_	—	28.5	15.0	1.90	Moderate
*SP*_0.2_ *R*_0_	—	25.0	15.0	1.67	Moderate
*SP*_0.4_ *R*_0_	—	24.5	15.0	1.63	Moderate
*SP*_0.6_ *R*_0_	—	23.0	15.0	1.53	Moderate

*V*_D_, *V*_P_ and *V*_K_ = equilibrium sediment volume of 10 g oven-dried soil, passing 425 μm sieve, in distilled water, PAM solution and kerosene, respectively; and FSR = *V*_D_/*V*_K_ or *V*_P_/*V*_K_.

**Table 6:** Summary of the compaction characteristics for the natural soil and various PAM-treated blends—*SP_x_R*_0_ where *x* = {0, 0.1, 0.2, 0.4, 0.6}.

Sample	*w*_opt_ (%)	*ρ*_dmax_ (Mg/m^3^)	*e*_min_^1^	*S*_R_^opt^ (%) ^2^
*SP*_0_ *R*_0_	20.24	1.62	0.702	79.59
*SP*_0.1_ *R*_0_	20.28	1.64	0.682	82.05
*SP*_0.2_ *R*_0_	21.06	1.66	0.662	87.82
*SP*_0.4_ *R*_0_	20.20	1.67	0.650	85.72
*SP*_0.6_ *R*_0_	20.72	1.70	0.622	91.88

^1^ Minimum void ratio, obtained by *e*_min_ = *G*_s_^S^ *ρ*_w_/*ρ*_dmax_ − 1; and ^2^ Optimum degree of saturation, obtained by *S*_R_^opt^ = *G*_s_^S^ *w*_opt_/*e*_min_.

**Table 7:** Summary of the stress–strain features for the natural soil and various PAM-treated samples—*SP_x_R*_0_ where *x* = {0, 0.1, 0.2, 0.4, 0.6}—at *T*_c_ = 1, 4, 7 and 14 d.

Sample	*T*_c_ (d)	*q*_u_ (kPa)	*η* (%) ^1^	*ε*_u_ (%) ^2^	*I*_D_^3^	*E*_50_ (MPa) ^4^	*E*_U_ (kJ/m^3^) ^5^	*E*_R_ (MPa) ^6^
*SP*_0_ *R*_0_	0	72.28	—	4.21	1.00	1.97	1.71	130.61
*SP*_0.1_ *R*_0_	1	96.87	34	4.62	1.10	2.83	2.76	151.63
*SP*_0.2_ *R*_0_	1	128.43	78	5.01	1.19	3.67	4.10	178.61
*SP*_0.4_ *R*_0_	1	83.18	15	6.81	1.62	1.39	3.17	139.92
*SP*_0.6_ *R*_0_	1	54.14	−25	8.02	1.90	0.75	2.37	115.10
*SP*_0.1_ *R*_0_	4	107.51	49	4.75	1.13	3.32	3.31	160.73
*SP*_0.2_ *R*_0_	4	141.23	95	5.20	1.24	4.28	4.89	189.55
*SP*_0.4_ *R*_0_	4	88.11	22	6.94	1.65	1.51	3.79	144.14
*SP*_0.6_ *R*_0_	4	62.12	−14	7.94	1.89	0.94	2.76	121.92
*SP*_0.1_ *R*_0_	7	117.16	62	4.67	1.11	3.76	3.65	168.98
*SP*_0.2_ *R*_0_	7	164.47	128	5.03	1.19	5.27	5.50	209.42
*SP*_0.4_ *R*_0_	7	109.43	51	6.88	1.63	2.03	4.29	162.37
*SP*_0.6_ *R*_0_	7	70.21	−3	8.11	1.93	1.08	3.14	128.84
*SP*_0.1_ *R*_0_	14	119.84	66	4.56	1.08	3.79	3.74	171.27
*SP*_0.2_ *R*_0_	14	168.34	133	4.87	1.16	5.29	5.30	212.73
*SP*_0.4_ *R*_0_	14	107.21	48	6.92	1.64	1.96	4.11	160.47
*SP*_0.6_ *R*_0_	14	68.87	−5	7.84	1.86	1.08	3.02	127.69

^1^ Percent change in the UCS (or *q*_u_); ^2^ Axial strain at failure; ^3^ *I*_D_ = *ε*_u_^S^/*ε*_u_^N^ where *ε*_u_^S^ = axial strain at failure for the stabilized sample, and *ε*_u_^N^ = axial strain at failure for the natural soil [63]; ^4^ *E*_50_ = secant modulus at 50% of the UCS (or *q*_u_) [66]; ^5^ *E*_U_ = area under the stress–strain curve up to the failure point [65]; and ^6^ Computed from *E*_R_ (psi) = 124 × *q*_u_ (psi) + 9980, as suggested by Thompson [64].

**Table 8:** Summary of the stress–strain features for the natural soil and various GR-blended samples—*SP_x_R_y_* where *x* = {0, 0.2}, and *y* = {0, 5, 10, 20, 30}.

Sample	*T*_c_ (d)	*q*_u_ (kPa)	*η* (%) ^1^	*ε*_u_ (%) ^2^	*I*_D_^3^	*E*_50_ (MPa) ^4^	*E*_U_ (kJ/m^3^) ^5^	*E*_R_ (MPa) ^6^
*SP*_0_ *R*_0_	0	72.28	—	4.21	1.00	1.97	1.71	130.61
*SP*_0_ *R*_5_	0	91.21	26	5.59	1.33	1.96	2.86	146.79
*SP*_0_ *R*_10_	0	98.61	36	7.61	1.81	1.51	4.17	153.12
*SP*_0_ *R*_20_	0	62.28	−14	8.78	2.09	0.72	2.83	122.06
*SP*_0_ *R*_30_	0	46.61	−36	9.58	2.28	0.47	2.19	108.66
*SP*_0.2_ *R*_0_	7	164.47	128	5.03	1.19	5.27	5.50	209.42
*SP*_0.2_ *R*_5_	7	208.94	189	5.82	1.38	4.54	7.05	247.44
*SP*_0.2_ *R*_10_	7	211.09	192	7.98	1.90	3.26	9.80	249.28
*SP*_0.2_ *R*_20_	7	106.18	47	9.01	2.14	1.25	5.26	159.59
*SP*_0.2_ *R*_30_	7	65.15	−10	9.22	2.19	0.66	3.14	124.51

^1^ Percent change in the UCS (or *q*_u_); ^2^ Axial strain at failure; ^3^ *I*_D_ = *ε*_u_^S^/*ε*_u_^N^ where *ε*_u_^S^ = axial strain at failure for the stabilized sample, and *ε*_u_^N^ = axial strain at failure for the natural soil [63]; ^4^ *E*_50_ = secant modulus at 50% of the UCS (or *q*_u_) [66]; ^5^ *E*_U_ = area under the stress–strain curve up to the failure point [65]; and ^6^ Computed from *E*_R_ (psi) = 124 × *q*_u_ (psi) + 9980, as suggested by Thompson [64].

**Table 9:** Summary of the soil reactivity features for the natural soil and various GR-blended samples—*SP_x_R_y_* where *x* = {0, 0.2}, and *y* = {0, 5, 10, 20, 30}.

Sample	*ε*_sh_ (%)	*ε*_sw_ (%)	*ψ*_o_ (pF)	*ψ*_sh_ (pF) ^1^	*ψ*_sw_ (pF) ^2^	∆*ψ* (pF)	*I*_ssc_ (%pF^−^^1^) ^3^	*I*_ssm_ (%pF^−1^) ^4^
*SP*_0_ *R*_0_	5.74	3.69	4.73	5.72	3.65	2.07	4.21	3.66
*SP*_0_ *R*_5_	5.34	3.03	4.75	5.69	3.60	2.09	3.81	3.28
*SP*_0_ *R*_10_	4.89	2.11	4.78	5.64	3.55	2.09	3.30	2.84
*SP*_0_ *R*_20_	2.93	1.77	4.77	5.61	3.33	2.28	2.12	1.67
*SP*_0_ *R*_30_	1.87	1.61	4.76	5.54	3.26	2.28	1.49	1.17
*SP*_0.2_ *R*_0_	3.52	1.98	4.74	5.74	3.63	2.11	2.51	2.14
*SP*_0.2_ *R*_5_	3.29	1.74	4.77	5.70	3.59	2.11	2.31	1.97
*SP*_0.2_ *R*_10_	2.62	1.54	4.81	5.67	3.53	2.14	1.88	1.58
*SP*_0.2_ *R*_20_	2.57	1.34	4.79	5.64	3.32	2.32	1.80	1.40
*SP*_0.2_ *R*_30_	1.31	1.14	4.77	5.55	3.25	2.30	1.04	0.82

^1^ Total suction upon completion of the core shrinkage test; ^2^ Total suction upon completion of the oedometer swell test; ^3^ Conventional shrink–swell index, obtained by ∆*ψ* = 1.8 pF; and ^4^ Modified shrink–swell index, obtained by actual ∆*ψ* measurements.

## Abbreviations

The following abbreviations are used in this manuscript.

A	Non-expansive
AMD	Acrylamide
AS	Australian standard
ASTM	American Society for Testing and Materials
CH	Clay with high plasticity
E	Extremely expansive
FSR	Free swell ratio
GR	Ground rubber
H	Highly expansive
M	Moderately expansive
PAM	Polyacrylamide
S	Slightly expansive
SEM	Scanning electron microscopy
SP	Poorly-graded sand
UCS	Unconfined compressive strength
USCS	Unified Soil Classification System
ZAV	Zero-air void

## Appendix A

Figure A1 shows a photograph of the anionic PAM, in its granular form, used in the present investigation.

Classification procedures for expansive soils based on the FSR, as proposed by Prakash and Sridharan [50], are summarized in Table A1.

**Table A1:** Classification procedures for expansive soils based on the FSR [50].

FSR ^1^	Degree of Expansivity	Dominant Clay Mineral
≤1	Negligible	Kaolinite
1–1.5	Low	Kaolinite and Montmorillonite
1.5–2	Moderate	Montmorillonite
2–4	High	Montmorillonite
>4	Very High	Montmorillonite

^1^ Ratio of equilibrium sediment volume of 10 g oven-dried soil, passing 425 μm sieve, in distilled water to that of kerosene.

Classification procedures for expansive soils based on the shrink–swell index *I*_ss_, as suggested by Seddon [59], are summarized in Table A2.

**Table A2:** Classification procedures for expansive soils based on the shrink–swell index *I*_ss_ [59].

*I*_ss_ (%pF^−1^) ^1^	Degree of Expansivity	*y*_s_ (mm) ^2^	Site Classification (AS 2870–11)
<0.8	Non-Expansive	< 10	Class A
0.8–1.7	Slightly Expansive	10–20	Class S
1.7–3.3	Moderately Expansive	20–40	Class M
3.3–5.8	Highly Expansive	40–70	Class H
>5.8	Extremely Expansive	> 70	Class E

^1^ Shrink–swell index, obtained by Equation (6); and ^2^ Free ground surface movement.

## References

[1] Soltani A., Taheri A., Deng A., Nikraz H. (2019). Tyre rubber and expansive soils: Two hazards, one solution. Proc. Inst. Civ. Eng. Constr. Mater..

[2] Jones L.D., Jefferson I., Burland J., Chapman T., Brown M., Skinner H. (2012). Expansive soils. ICE Manual of Geotechnical Engineering: Volume I.

[3] Soltani A., Deng A., Taheri A., Mirzababaei M., Vanapalli S.K. (2019). Swell–shrink behavior of rubberized expansive clays during alternate wetting and drying. Minerals.

[4] Soltani A., Deng A., Taheri A., Mirzababaei M. (2018). Rubber powder–polymer combined stabilization of South Australian expansive soils. Geosynth. Int..

[5] Winterkorn H.F., Pamukcu S., Fang H.Y. (1991). Soil stabilization and grouting. Foundation Engineering Handbook.

[6] Soltani A., Taheri A., Khatibi M., Estabragh A.R. (2017). Swelling potential of a stabilized expansive soil: A comparative experimental study. Geotech. Geol. Eng..

[7] Al-Omari R.R., Hamodi F.J. (1991). Swelling resistant geogrid—A new approach for the treatment of expansive soils. Geotext. Geomembranes.

[8] Tang C.S., Shi B., Zhao L.Z. (2010). Interfacial shear strength of fiber reinforced soil. Geotext. Geomembranes.

[9] Mirzababaei M., Miraftab M., Mohamed M., McMahon P. (2013). Unconfined compression strength of reinforced clays with carpet waste fibers. J. Geotech. Geoenviron. Eng..

[10] Estabragh A.R., Rafatjo H., Javadi A.A. (2014). Treatment of an expansive soil by mechanical and chemical techniques. Geosynth. Int..

[11] Phanikumar B.R., Singla R. (2016). Swell–consolidation characteristics of fibre-reinforced expansive soils. Soils Found..

[12] Wang Y.X., Guo P.P., Ren W.X., Yuan B.X., Yuan H.P., Zhao Y.L., Shan S.B., Cao P. (2017). Laboratory investigation on strength characteristics of expansive soil treated with jute fiber reinforcement. Int. J. Geomech..

[13] Soltani A., Deng A., Taheri A. (2018). Swell‒compression characteristics of a fiber-reinforced expansive soil. Geotext. Geomembranes.

[14] Miller G.A., Azad S. (2000). Influence of soil type on stabilization with cement kiln dust. Constr. Build. Mater..

[15] Dash S.K., Hussain M. (2012). Lime stabilization of soils: Reappraisal. J. Mater. Civ. Eng..

[16] Estabragh A.R., Pereshkafti M.R.S., Parsaei B., Javadi A.A. (2013). Stabilised expansive soil behaviour during wetting and drying. Int. J. Pavement Eng..

[17] Keramatikerman M., Chegenizadeh A., Nikraz H. (2016). Effect of GGBFS and lime binders on the engineering properties of clay. Appl. Clay Sci..

[18] Sharma A.K., Sivapullaiah P.V. (2016). Ground granulated blast furnace slag amended fly ash as an expansive soil stabilizer. Soils Found..

[19] Zhao Y., Soltani A., Taheri A., Karakus M., Deng A. (2019). Application of slag–cement and fly ash for strength development in cemented paste backfills. Minerals.

[20] Estabragh A.R., Namdar P., Javadi A.A. (2012). Behavior of cement-stabilized clay reinforced with nylon fiber. Geosynth. Int..

[21] Olgun M. (2013). The effects and optimization of additives for expansive clays under freeze–thaw conditions. Cold Reg. Sci. Technol..

[22] Shahbazi M., Rowshanzamir M., Abtahi S.M., Hejazi S.M. (2017). Optimization of carpet waste fibers and steel slag particles to reinforce expansive soil using response surface methodology. Appl. Clay Sci..

[23] Mirzababaei M., Arulrajah A., Horpibulsuk S., Soltani A., Khayat N. (2018). Stabilization of soft clay using short fibers and poly vinyl alcohol. Geotext. Geomembr..

[24] Zhang J., Soltani A., Deng A., Jaksa M.B. (2019). Mechanical performance of jute fiber-reinforced micaceous clay composites treated with ground-granulated blast-furnace slag. Materials.

[25] Li J., Saberian M., Nguyen B.T. (2018). Effect of crumb rubber on the mechanical properties of crushed recycled pavement materials. J. Environ. Manag..

[26] Yadav J.S., Tiwari S.K. (2017). Effect of waste rubber fibres on the geotechnical properties of clay stabilized with cement. Appl. Clay Sci..

[27] Soltani A., Deng A., Taheri A., Mirzababaei M., Nikraz H. (2019). Interfacial shear strength of rubber-reinforced clays: A dimensional analysis perspective. Geosynth. Int..

[28] Edil T., Bosscher P. (1994). Engineering properties of tire chips and soil mixtures. Geotech. Test. J..

[29] Foose G.J., Benson C.H., Bosscher P.J. (1996). Sand reinforced with shredded waste tires. J. Geotech. Eng..

[30] Al-Tabbaa A., Blackwell O., Porter S.A. (1997). An investigation into the geotechnical properties of soil–tyre mixtures. Environ. Technol..

[31] Lee J.H., Salgado R., Bernal A., Lovell C.W. (1999). Shredded tires and rubber sand as lightweight backfill. J. Geotech. Geoenviron. Eng..

[32] Zornberg J.G., Cabral A.R., Viratjandr C. (2004). Behaviour of tire shred–sand mixtures. Can. Geotech. J..

[33] Patil U., Valdes J.R., Evans T.M. (2011). Swell mitigation with granulated tire rubber. J. Mater. Civ. Eng..

[34] Trouzine H., Bekhiti M., Asroun A. (2012). Effects of scrap tyre rubber fibre on swelling behaviour of two clayey soils in Algeria. Geosynth. Int..

[35] Kalkan E. (2013). Preparation of scrap tire rubber fiber–silica fume mixtures for modification of clayey soils. Appl. Clay Sci..

[36] Cabalar A.F., Karabash Z., Mustafa W.S. (2014). Stabilising a clay using tyre buffings and lime. Road Mater. Pavement Des..

[37] Signes C.H., Garzón-Roca J., Fernández P.M., Torre M.E.G., Franco R.I. (2016). Swelling potential reduction of Spanish argillaceous marlstone Facies Tap soil through the addition of crumb rubber particles from scrap tyres. Appl. Clay Sci..

[38] Soltani A., Deng A., Taheri A., Sridharan A. (2019). Swell–shrink–consolidation behavior of rubber-reinforced expansive soils. Geotech. Test. J..

[39] Inyang H.I., Bae S., Mbamalu G., Park S. (2007). Aqueous polymer effects on volumetric swelling of Na–montmorillonite. J. Mater. Civ. Eng..

[40] Kim S., Palomino A.M. (2009). Polyacrylamide-treated kaolin: A fabric study. Appl. Clay Sci..

[41] Mirzababaei M., Yasrobi S.S., Al-Rawas A.A. (2009). Effect of polymers on swelling potential of expansive soils. Proc. Inst. Civ. Eng. Ground Improv..

[42] Yazdandoust F., Yasrobi S.S. (2010). Effect of cyclic wetting and drying on swelling behavior of polymer-stabilized expansive clays. Appl. Clay Sci..

[43] Iyengar S.R., Masad E., Rodriguez A.K., Bazzi H.S., Little D., Hanley H.J.M. (2013). Pavement subgrade stabilization using polymers: Characterization and performance. J. Mater. Civ. Eng..

[44] Onyejekwe S., Ghataora G.S. (2015). Soil stabilization using proprietary liquid chemical stabilizers: Sulphonated oil and a polymer. Bull. Eng. Geol. Environ..

[45] Georgees R.N., Hassan R.A., Evans R.P. (2017). A potential use of a hydrophilic polymeric material to enhance durability properties of pavement materials. Constr. Build. Mater..

[46] Bian X., Zeng L., Deng Y., Li X. (2018). The role of superabsorbent polymer on strength and microstructure development in cemented dredged clay with high water content. Polymers.

[47] Soltani-Jigheh H., Bagheri M., Amani-Ghadim A.R. (2019). Use of hydrophilic polymeric stabilizer to improve strength and durability of fine-grained soils. Cold Reg. Sci. Technol..

[48] Soltani A., Deng A., Taheri A., Mirzababaei M. (2019). A sulphonated oil for stabilisation of expansive soils. Int. J. Pavement Eng..

[49] Sridharan A., Nagaraj H.B., Prakash K. (1999). Determination of the plasticity index from flow index. Geotech. Test. J..

[50] Prakash K., Sridharan A. (2004). Free swell ratio and clay mineralogy of fine-grained soils. Geotech. Test. J..

[51] Seybold C.A. (1994). Polyacrylamide review: Soil conditioning and environmental fate. Commun. Soil Sci. Plant Anal..

[52] Barvenik F.W. (1994). Polyacrylamide characteristics related to soil applications. Soil Sci..

[53] Lu J.H., Wu L., Letey J. (2002). Effects of soil and water properties on anionic polyacrylamide sorption. Soil Sci. Soc. Am. J..

[54] Graber E.R., Fine P., Levy G.J. (2006). Soil stabilization in semiarid and arid land agriculture. J. Mater. Civ. Eng..

[55] Orts W.J., Sojka R.E., Glenn G.M., Gross R.A. (1999). Preventing soil erosion with polymer additives. Polym. News.

[56] Orts W.J., Roa-Espinosa A., Sojka R.E., Glenn G.M., Imam S.H., Erlacher K., Pedersen J.S. (2007). Use of synthetic polymers and biopolymers for soil stabilization in agricultural, construction, and military applications. J. Mater. Civ. Eng..

[57] Soltani A., Deng A., Taheri A., Sridharan A. (2019). Consistency limits and compaction characteristics of clay soils containing rubber waste. Proc. Inst. Civ. Eng. Geotech. Eng..

[58] Fityus S.G., Cameron D.A., Walsh P.F. (2005). The shrink swell test. Geotech. Test. J..

[59] Seddon K.D., Peck W.A., Neilson J.L., Olds R.J., Seddon K.D. (1992). Reactive soils. Proceedings of the Seminar on Engineering Geology of Melbourne.

[60] Zou J. (2015). Assessment of the Reactivity of Expansive Soil in Melbourne Metropolitan Area. Master’s Thesis.

[61] Soltani A., Estabragh A.R., Taheri A., Deng A., Meegoda J.N. (2018). Experiments and dimensional analysis of contaminated clay soils. Environ. Geotech..

[62] Mitchell J.K., Soga K. (2005). Fundamentals of Soil Behavior.

[63] Park S.S. (2011). Unconfined compressive strength and ductility of fiber-reinforced cemented sand. Constr. Build. Mater..

[64] Thompson M.R. (1986). Mechanistic Design Concepts for Stabilized Base Pavements, Technical Report UILU-ENG-86-2008.

[65] Maher M.H., Ho Y.C. (1994). Mechanical properties of kaolinite/fiber soil composite. J. Geotech. Eng..

[66] Radovic M., Lara-Curzio E., Riester L. (2004). Comparison of different experimental techniques for determination of elastic properties of solids. Mater. Sci. Eng. A.

[67] Theng B.K.G. (1982). Clay–polymer interactions: Summary and perspectives. Clays Clay Miner..

[68] Ben-Hur M., Malik M., Letey J., Mingelgrin U. (1992). Adsorption of polymers on clays as affected by clay charge and structure, polymers properties, and water quality. Soil Sci..

[69] Greenland D.J. (1963). Adsorption of polyvinyl alcohols by montmorillonite. J. Colloid Sci..

[70] Letey J. (1994). Adsorption and desorption of polymers on soil. Soil Sci..

[71] Laird D.A. (1997). Bonding between polyacrylamide and clay mineral surfaces. Soil Sci..

[72] Luckham P.F., Rossi S. (1999). The colloidal and rheological properties of bentonite suspensions. Adv. Colloid Interface Sci..

